# Development and validation of a predictive model to forecast the cerebrovascular risk burden in older people using the NACC and ROSMAP dataset

**DOI:** 10.1002/alz.70543

**Published:** 2025-08-12

**Authors:** Chenyin Chu, Yihan Wang, Liwei Ma, Liang Jin, Yijun Pan

**Affiliations:** ^1^ School of Translational Medicine Monash University Melbourne Victoria Australia; ^2^ Florey Department of Neuroscience and Mental Health University of Melbourne Victoria Australia

**Keywords:** autoscore algorithm, cerebrovascular disease burden, machine learning, risk stratification, vascular dementia

## Abstract

**INTRODUCTION:**

The integrity of the brain's vascular system is vital for neuronal health. Cerebrovascular microbleeds, microinfarcts, and infarcts (defined as cerebrovascular disease burden) contribute to stroke and cognitive impairment. Here, we developed and validated the first model, the Cerebrovascular Disease Burden Risk Score (CDBRS) for individualized risk prediction.

**METHODS:**

Leveraging the AutoScore and AutoScore‐Ordinal algorithms, the CDBRS was developed using National Alzheimer's Coordinating Center (NACC) data and externally validated on Religious Orders Study and Memory and Aging Project (ROSMAP) data. The CDBRS was evaluated using mean area under the receiver operating characteristic curve (mAUC), Harrell's generalized c‐index, sensitivity, and specificity.

**RESULTS:**

The CDBRS achieved a promising predictive performance and outperformed the respective baseline models. The performance of the CDBRS remains good on external validation.

**DISCUSSION:**

CDBRS can possibly aid in identifying individuals with high risks of cerebrovascular microbleeds, microinfarcts, and infarcts, to enable closer monitoring and personalized management. Further validation of CDBRS in a diverse population is warranted.

**Highlights:**

- Microbleeds, microinfarcts, and infarcts often precede clinical stroke and vascular dementia.
- We developed and validated the Cerebrovascular Disease Burden Risk Score (CDBRS), a new and interpretable model for stratifying the risk of microbleeds, microinfarcts, and infarcts in older adults.
- This tool developed using National Alzheimer's Coordinating Center (NACC) participants data achieved promising predictive performance in Religious Orders Study and Memory and Aging Project (ROSMAP) participants.
- The robust predictive performance warrants its clinical validation is prospective studies.

## 1 BACKGROUND

The global health landscape is increasingly challenged by age‐related conditions, particularly those affecting the brain's vascular system and cognitive function.^1^, ^2^ The integrity of the brain's vascular system is paramount for neuronal health and overall brain function.^3^ A key component of this challenge lies in small vessel disease (SVD), a pervasive condition that affects the brain's microvasculature and contributes significantly to ischemic stroke and is also implicated in hemorrhagic stroke and vascular cognitive impairment.^4^

SVD manifests through a variety of subtle yet damaging pathologies, including microbleeds, microinfarcts, and infarcts.^5^ Cerebral microbleeds are small, chronic lesions caused by the rupture of tiny blood vessels, leading to localized areas of hemorrhage.^6^ They are linked to an increased risk of both hemorrhagic and ischemic stroke and serve as indicators of underlying vascular fragility.^7^, ^8^ Microinfarcts, on the other hand, are tiny areas of tissue death resulting from the obstruction of small blood vessels, often due to thrombosis or embolism.^9^, ^10^ While individually microscopic, their cumulative effect can significantly impair the function of critical brain regions involved in memory and executive function, particularly in older people.^11^, ^12^, ^13^ Infarcts, which refer to larger areas of tissue death caused by a complete blockage of blood flow to a region of the brain, are more clinically obvious but often share underlying pathophysiological mechanisms with microbleeds and microinfarcts.^14^ Due to their often‐subclinical nature, they frequently go undetected until significant and often irreversible damage occurs.

Predicting the risk of cerebrovascular microbleeds, microinfarcts, and infarcts is crucial for early intervention and prevention of long‐term complications, which allows for timely management of modifiable vascular risk factors like hypertension, diabetes, and hyperlipidemia. Those interventions may enable proactive strategies to reduce the likelihood of ischemic and hemorrhagic strokes, and vascular dementia.^15^ Furthermore, it can serve as a screening tool to identify patients that require more focused monitoring through imaging techniques, ensuring that any changes in brain health are caught early, facilitating prompt adjustments in treatment and care.

Currently, there is no model that allows the prediction of risk of cerebrovascular microbleeds, microinfarcts, and infarcts. To address this, we employed a machine learning and statistical modeling approach to develop and validate the Cerebrovascular Disease Burden Risk Score (CDBRS) model. By providing clinicians with a stratified cerebrovascular risk for each patient, the CDBRS seeks to facilitate more informed, personalized decision‐making regarding health management, ultimately aiming to prevent complications from cerebrovascular diseases by early intervention.

## 2 METHODS

### 2.1 Data sources and ethics

This study utilized data from two primary sources: the U.S. National Alzheimer's Coordinating Center (NACC) and the Religious Orders Study (ROS) and Memory and Aging Project (MAP) (ROSMAP). The NACC, supported by the U.S. National Institute on Aging/National Institutes of Health,^16^ has been collecting data from Alzheimer's Disease Centers (ADCs) since 1999. This study specifically drew from the NACC's Uniform Data Set (UDS) and its neuropathology dataset. The UDS provides comprehensive information on participants' cognitive status, demographics, medical and family history, and clinical details related to their cognitive, motor, functional, and neuropsychiatric health. Trained clinicians prospectively gather and record the UDS data annually. Data from the NACC was accessed via its official website https://naccdata.org/. The ROSMAP study combines data from two longitudinal, epidemiologic clinicopathological studies: the ROS, which began in 1994, and the MAP, initiated in 1997.^17^ Both studies recruit participants without known dementia in the United States to investigate aging and dementia. Rush University Medical Centre's Institutional Review Board approved the ROSMAP study, and all participants provided informed consent. ROSMAP data were obtained from its dedicated website https://www.radc.rush.edu. The current study performed secondary data analysis of the de‐identified patient data. Institution approval has been obtained from Monash University (Project ID 48289).

### 2.2 Participants and features

Model development was leveraged on the NACC data. Cerebrovascular disease burden risk was evaluated by using three binary neuropathological features—microbleeds, microinfarcts, and infarcts. 3,815 NACC participants who have all these features recorded were identified from the dataset. During data pre‐processing, features (potential predictors) with more than 10% missing data were excluded from analysis. The remaining features are listed in eTable 1. The dataset contains many features but a relatively modest sample size, and therefore a complete‐case approach would have discarded participants whose missing data occurred in variables unrelated to the model.^18^ We conducted a random forest variable importance analysis on the full cohort of 3,815 NACC participants. We calculated importance scores for every candidate features and retained only features with a score > 5 for downstream modelling. Participants who still had missing values in any of these retained features were removed. This resulted in a final cohort of 2,047 NACC participants for the study. Following the same procedure, we identified 877 ROSMAP participants, and their data was used for external validation of the CDBRS.

### 2.3 CDBRS model development

The predicted outcome in our study is the cerebrovascular disease burden risk, classified as low, medium, and high, which is an ordinal outcome. Low is defined as having no cerebrovascular microbleeds, microinfarcts, and infarcts, medium is defined as having one of these pathologies, and high is defined as having more than one of these pathologies. An alternative predicted outcome is the presence or absence of cerebrovascular disease burden risk, characterized by whether the individual has any of cerebrovascular microbleeds, microinfarcts, and infarcts or not. The development of the CDBRS mainly leverages the AutoScore and AutoScore‐Ordinal algorithms and their accompanying R packages. An architecture framework has been prepared to illustrate the model construction process (eFigure 1). Detailed methods of the model development are available in the Supplementary Materials.

**RESEARCH IN CONTEXT**

1. **Systematic review**: Our comprehensive literature review across PubMed, Google Scholar, and Web of Science revealed a notable gap: while epidemiological studies have identified risk factors for cerebrovascular disease, there is currently no readily available machine learning model capable of predicting cerebrovascular disease risk in living patients. Existing models do not adequately address this clinical need.
2. **Interpretation**: To address this unmet need, we developed and validated the Cerebrovascular Disease Burden Risk Score (CDBRS), a new predictive tool for cerebrovascular disease burden. The CDBRS model was constructed using the AutoScore algorithm, which automates the development of interpretable clinical scoring models. We observed promising prediction performance for CDBRS among National Alzheimer's Coordinating Center (NACC) participants, and critically, this performance was reproducible upon external validation with Religious Orders Study and Memory and Aging Project (ROSMAP) participants.
3. **Future directions**: The CDBRS holds substantial potential to facilitate more informed and personalized decision‐making in health management, ultimately aiming to prevent complications from cerebrovascular diseases through early detection and intervention. Its potential clinical utility and possible impact on patient outcomes warrant further validation in prospective studies.

The pre‐processed NACC data was randomly partitioned into a training set (70%) and an independent test set (30%) and further processed through the AutoScore/AutoScore‐Ordinal modules: (1) Feature ranking**—**Features were ranked by their importance using a random forest algorithm specifically designed for binary/ordinal classification tasks. (2) Variable transformation—Continuous variables were categorized to minimize the impact of non‐linearity and outliers on model performance, which is commonly used in medical research.^19^ (3) Score derivation—Variable weights were calculated using the cumulative link model, a robust regression method for ordinal outcome.^20^ These weights were then normalized and rescaled to produce scores for each variable. (4) Parsimony analysis—A parsimony analysis with a 10‐fold cross‐validation identified the optimal subset of variables that balances model simplicity and predictive power. (5) Clinical fine‐tuning—Algorithm‐generated cutoffs were aligned with established clinical thresholds as per guidelines. (6) Model evaluation—The final scoring system was tested on an independent hold‐out dataset to assess discrimination and calibration. Training set was used for Modules 1—5, and test set was used for Module 6.

Two complementary scoring tools were derived. CDBRS‐3 classifies cerebrovascular disease burden risk into three ordered categories (low, medium, high), while CDBRS‐2 provides a simpler binary flag that separates individuals with no burden from those with any level of burden. Designing both versions offers clinicians flexibility: CDBRS‐3 supports granular staging, whereas CDBRS‐2 facilitates rapid screening. Both models broaden potential use‐cases and lay the groundwork for future studies to pinpoint the most effective risk‐stratification strategy for specific clinical settings.

### 2.4 CDBRS model evaluation

The predictive accuracy of CDBRS‐3 was quantified on the independent test set with two metrics. First, we calculated the mean area under the receiver operating characteristic curve (mAUC) because the outcome contains three ordered categories, the task was decomposed into two one‐versus‐rest binary sub‐problems; an AUC was obtained for each sub‐problem and their arithmetic mean reported as mAUC.^21^ Second, we applied Harrell's generalized c‐index, which expresses the proportion of all observation pairs whose predicted risk ordering coincides with the observed ordering.^22^ For both metrics, a score of 0.5 indicates chance‐level performance, whereas 1.0 denotes perfect discrimination. To express sampling uncertainty, we derived 95% bootstrap confidence intervals around each point estimate. The binary CDBRS‐2 model was evaluated with sensitivity and specificity ‐ metrics that directly characterize true‐positive and true‐negative detection rates in two‐class settings.

For performance evaluation, we compared the CDBRS models against several baseline models. CDBRS‐3 was compared with three proportional‐odds models (POM): POM‐A with LASSO method for feature selection, POM‐B with a stepwise feature selection, and POM‐C with the same features as CDBRS‐3. CDBRS‐2 was compared with a logistic regression model whose predictors were selected through a random forest‐based feature selection (logistic‐base), which is commonly used in binary classification.

### 2.5 External validation on ROSMAP data

To test generalizability of the developed CDBRS models, we externally validate them in 877 ROSMAP participants. As the two cohorts differ, we restricted the analysis to features shared by both datasets. We manually aligned the feature set to enable cross‐cohort assessment. Moreover, to evaluate the model's predictive stability or directionality, we also test the performance of the model with these aligned features on the test set of the NACC participants. After that, the model was externally evaluated with ROSMAP participant data. The prediction performance was evaluated against clinical diagnoses, and the mAUC and Harrell's generalized c‐index was presented.

### 2.6 Epidemiology analysis

To complement machine learning, epidemiology analysis was performed. Both binary and multinomial logistic regression models were used to evaluate the targeted associations. We assessed the associations between participant characteristics and the presence of hemorrhages/microbleeds, microinfarcts, and grossly observed old infarcts; these three outcomes were treated as dichotomous variables. All regression models were adjusted for age (in years), sex, and apolipoprotein E (APOE) ε4 carrier status (yes/no). The threshold for statistical significance was set at *p* < 0.05.

### 2.7 Statistical analysis

All pre‐processing and statistical analyses were carried out in Python 3.9, R 3.5.3 (RStudio version 12.0+369), and Stata (version 17.0). Model development used the AutoScore package,^23^, ^24^ which facilitates the streamlined creation of point‐based clinical scoring models for outcome prediction. The 95% confidence intervals (CIs) were estimated from 10 bootstrap resamples. The performance of CDBRS model was compared with its corresponding baseline model using DeLong's non‐parametric test, with two‐sided *p* < 0.05 considered statistically significant.

## 3 RESULTS

### 3.1 Participant characteristics

The CDBRS models were developed using data collected from 2,047 NACC participants and externally validated on 877 ROSMAP participants. At baseline, NACC participants had a mean age of 74.6 ± 9.4 (mean ± SD) years; 48.5% were female, and 43.9% carried at least one APOE ε4 allele. Educational attainment averaged 15.8 ± 2.9 years. Cerebrovascular disease burden risk was classified as low for 1,384, medium for 512, and high for 151 participants. The ROSMAP participants had a mean age of 81.95 ± 6.6 years, with a higher proportion of female (71.7%), and lower prevalence of APOE ε4 carriage (24.1%) compared to the NACC participants. Educational attainment averaged 14.7 ± 2.9 years. Cerebrovascular disease burden risk was classified as low for 452, medium for 307, and high for 145 participants. A summary of demographic and clinical variables is provided in Table 1.

**TABLE 1:** Participant characteristics.

Features	NACC (*n* = 2,047)	ROSMAP (*n* = 877)
Age (years)	74.58 (9.41)	81.95 (6.61)
Sex	Male: 1055; Female: 992	Male: 248; Female: 629
Body mass index	26.48 (4.57)	27.01 (5.12)
Education (years)	15.84 (2.91)	14.66 (2.90)
APOE genotype	ε3/ε3:971; ε4/ε3:663; ε3/ε2:168; ε4/ε4:165; ε4/ε2:70; ε2/ε2:10	ε3/ε3:539; ε4/ε3:173; ε3/ε2:120; ε4/ε4:17; ε4/ε2:21; ε2/ε2:7
Time after baseline (years)	6.64 (3.59)	7.18 (4.46)
Diastolic blood pressure (mmHg)	134.99 (18.07)	135.42 (17.60)
Systolic blood pressure (mmHg)	74.50 (10.03)	73.05 (11.09)
Heart rate (per minutes)	67.00 (10.53)	NA
Boston Naming Test (30‐items)	24.72 (5.69)	NA
Mini‐Mental State Examination (MMSE)	26.10 (4.11)	27.18 (3.63)
Arteriolosclerosis	None: 329; mild: 732; moderate: 706; severe: 280	None: 283; mild: 305; moderate: 216; severe: 73
Cerebrovascular disease burden	Low: 1384; medium: 512; high: 151	Low: 452; medium: 307; high: 145

*Note*: Data are presented as mean (SD). All data were collected at baseline except neuropathology data. Neuropathological evaluations were conducted by individual Alzheimer's Disease Centers (ADCs) following their own protocols, which may vary but are in accordance with the consensus guidelines. The results were entered into the NACC database using a standardized NACC form. According to the NACC coding guidelines, severity was based on the neuropathologist's overall assessment of severity, rather than on the condition of an individual vessel.

Abbreviations: APOE, apolipoprotein E; NACC, National Alzheimer's Coordinating Center; ROSMAP, Religious Orders Study and Memory and Aging Project.

### 3.2 Outcomes of CDBRS‐3 model construction and validation

**FIGURE 1:**
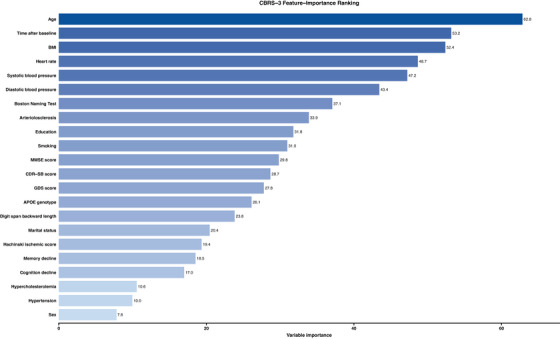
Feature ranking. This plot illustrates the relative importance of features derived from a random forest‐based feature selection. The x‐axis quantifies each feature's importance value, while the y‐axis lists the analyzed features. The numbers at the end of the bars indicate the exact importance value of each feature.

Among the initial 85 features, those with > 10% missing data or an importance value < 5 were discarded, leaving 25 features for model development. We employed the random forest algorithm to rank the importance of these features (Figure **1**). Age at baseline emerged as the single most important feature to predict cerebrovascular disease burden risk, mirroring the well‐known age‐related increase in disease risk.^25^ Body mass index (BMI) and follow‐up years followed closely. Cardiovascular factors (systolic and diastolic blood pressure, heart rate, arteriolosclerosis), neuropsychological measures (Boston Naming, Mini‐Mental State Examination [MMSE]), and APOE genotype occupied the next tier of importance. Additional demographic and clinical factors such as education, marital status, depressive symptoms, Hachinski score, smoking frequency, hypercholesterolemia, and hypertension showed moderate yet meaningful importance.

**FIGURE 2:**
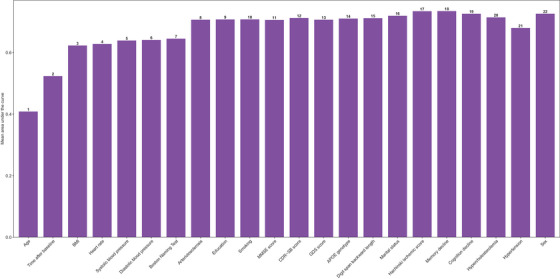
Parsimony plot for CDBRS‐3. This parsimony plot shows the relationship between the number of features used by the CDBRS‐3 model and its performance, measured by average mAUC values within the 10‐fold cross validation. The numbers on the bars indicate the cumulative count of features included at each step. Taller bars signify superior model performance. Abbreviations: AUC, area under the receiver operating characteristics curve; APOE, Apolipoprotein E; BMI, Body Mass Index; CDBRS, Cerebrovascular Disease Burden Risk Score; CDR‐SB, Clinical Dementia Rating ‐ Sum of Boxes; MMSE, Mini‐Mental State Examination; GDS, Geriatric Depression Scale.

Parsimony analysis was conducted to identify the optimal number of features required for the optimal performance of CDBRS‐3 (Figure 2). The model's mAUC rose sharply from 0.41 to 0.62 after the first three features being included. Incorporating the fourth through seventh features yielded only modest fluctuation (mAUC 0.62 to 0.64). The mAUC rose to 0.71 when eight features were used. The improvement in model performance was limited when additional features were added. Therefore, we decided to include eight features for the CDBRS‐3, considering the balance between model simplicity and predictive power.

After identifying the most relevant variables, we applied a cumulative log‐link regression model to assign scores for each category or interval of the selected features (Table 2, left panel). On the test set, the CDBRS‐3 achieved a mAUC of 0.70 (95% CI, 0.68–0.73) and Harrell's generalized c‐index of 0.69 (95% CI, 0.67–0.72) before fine tuning. To improve clinical utility, we refined the intervals for each feature as per their clinical norms. BMI categories were adjusted to as [0, 18.5), [18.5, 25), [25, 30), and > 30^26^; diastolic blood pressure intervals were < 60, [60, 80), [80, 90), and ≥90, systolic blood pressure intervals were < 120, [120, 150), and ≥150^27^; age intervals were < 60, [60, 80), and ≥80, and heart rate intervals were < 60, [60, 100), and ≥100. The revised scores for each feature following fine tuning are presented in right panel of Table 2.

**TABLE 2:** Score table for CDBRS‐3 model.

Feature	Before fine‐tuning		After fine‐tuning	
	Interval	Score	Interval	Score
Time after baseline	<3.32	0	<1.18	0
	[3.32,9.68)	1	[1.18,3.32)	1
	[9.68,13)	3	[3.32,9.68)	2
	≥13	4	[9.68,13)	3
			≥13	8
Body mass index*	<20	0	<18.5	10
	[20,22.5)	3	[18.5,25)	0
	[22.5,29.7)	7	[25,30)	13
	[29.7,35.4)	9	≥30	14
	≥35.4	7		
Blood pressure (systolic)*	<107	1	<120	0
	[107,120)	0	[120,150)	5
	[120,150)	3	≥150	6
	[160,167)	9		
	≥167	2		
Age*	<58	0	<60	0
	[58,66)	8	[60,80)	17
	[66,83)	17	≥80	26
	[83,88.4)	23		
	≥88,4	32		
Heart rate*	<51	2	<60	0
	[51,60)	3	[60,100)	6
	[60,76)	4	≥100	10
	[76,84)	0		
	≥84	7		
Blood pressure (diastolic)*	<66	4	<60	4
	[66,82)	1	[60,90)	3
	[82,90)	0	≥90	0
	≥90	1		
Boston Naming Test	<13	0	<22	0
	[13,22)	13	[22,26)	1
	≥22	12	≥26	2
Arteriolosclerosis	None	0	None	0
	Mild	8	Mild	9
	Moderate	16	Moderate	18
	Severe	23	Severe	26

*Note*: *fine‐tuning based on clinical norms.

Abbreviation: CDBRS‐3, Cerebrovascular Disease Burden Risk Score.

**FIGURE 3 alz70543-fig-0003:**
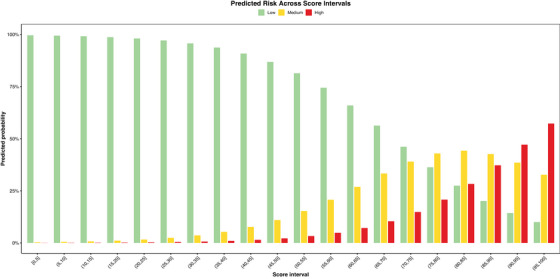
Score intervals and risk probability for CDBRS‐3. This plot displays the CDBRS‐3 score intervals on the x‐axis and the probability of risk on the y‐axis, represented by color‐coded bars (low: green, medium: yellow, high: red). As the score increases, so does the probability of a higher risk. CDBRS‐3, Cerebrovascular Disease Burden Risk Score.

After fine‐tuning, the CDBRS‐3 model retained comparable performance on the test set, with a mAUC of 0.73 (95% CI, 0.71–0.75) and Harrell's generalized c‐index of 0.72 (95% CI, 0.70–0.75). We subsequently mapped the scores to risk: low (0–75), medium (75–90), and high (90–100). A risk plot (Figure 3) illustrated the relationship between score intervals and the probability of cerebrovascular disease burden, revealing a clear trend of increasing disease burden with higher scores. Furthermore, the calibration plot (eFigure 2) reinforces the CDBRS‐3 model's accuracy, demonstrating the observed and predicted risks for each five‐point score interval closely align with the perfect calibration line. These findings underscore the CDBRS‐3 model's ability to effectively stratify the disease burden.

We also conducted a sensitivity analysis on the number of features selected to assess the robustness of our model's performance on the test set. Based on the parsimony analysis in Figure 2, we chose the CDBRS‐3 model with 9, 10, 11, and 12 features, which exhibited a performance similar to the 8‐feature model while balancing accuracy and complexity. The performance of the CDBRS‐3 model with these feature counts is shown in eTable 2. We observed that using more than eight features offered no significant improvement while increased model complexity.

### 3.3 Outcomes of CDBRS‐2 model construction and validation

The CDBRS‐2 model was generated with the AutoScore algorithm to distinguish individuals with low from those with medium or high risk. The selected features (eFigure 3) were almost identical to those for the CDBRS‐3 model, differing only slightly in their importance ranking. Parsimony analysis (eFigure 4) showed that seven features were sufficient for optimal performance, and the finalized scoring table is presented in eTable 3. When evaluated on the test set, the CDBRS‐2 model achieved an AUC of 0.72 (95% CI, 0.69–0.74), sensitivity of 0.73 (95% CI, 0.68–0.76), and specificity of 0.70 (95% CI, 0.67–0.72) using the optimized cutoff score of 59.

### 3.4 Comparison between the baseline models and CDBRS

Table 3A summarizes the performance comparison between the two CDBRS models and their respective baseline models. CDBRS‐3 consistently outperformed its baselines: POM‐A (Harrell's generalized c‐index: 0.54 [95% CI, 0.51–0.56]; mAUC: 0.57 [95% CI, 0.54–0.60]), POM‐B (Harrell's generalized c‐index: 0.56 [95% CI, 0.52–0.59]; mAUC: 0.54 [95% CI, 0.51–0.57]), and POM‐C (Harrell's generalized c‐index: 0.65 [95% CI, 0.63–0.68]; mAUC‐ROC: 0.64 [95% CI, 0.62–0.67]). Similarly, the CDBRS‐2 showed a better performance than the logistic‐base model (AUC: 0.60 [95% CI, 0.56–0.65]; sensitivity: 0.74 [95% CI: 0.70–0.78]; specificity: 0.40 [95% CI, 0.33–0.48]). These results indicate that the CDBRS models performed more effectively than their baseline counterparts.

**TABLE 3 alz70543-tbl-0003:** Model performance evaluation.

A: Comparison between CDBRS models and baseline models					
**Model**	**Mean AUC‐ROC**	**Generalized c‐index**	**AUC‐ROC**	**Specificity**	**Sensitivity**
CDBRS‐3	0.73 (95% CI, 0.71–0.75)	0.72 (95% CI, 0.70–0.75)	NA	NA	NA
POM‐A	0.57 (95% CI, 0.54–0.60)	0.54 (95% CI, 0.51–0.56)	NA	NA	NA
POM‐B	0.54 (95% CI, 0.51–0.57)	0.56 (95% CI, 0.52–0.59)	NA	NA	NA
POM‐C	0.64 (95% CI, 0.62–0.67)	0.65 (95% CI, 0.63–0.68)	NA	NA	NA
CDBRS‐2	NA	NA	0.72 (95% CI: 0.69–0.74)	0.70 (95% CI: 0.67–0.72)	0.73 (95% CI: 0.68–0.76)
Logistic‐selected	NA	NA	0.60 (95% CI 0.56–0.65)	0.74 (95% CI 0.70–0.78)	0.40 (95% CI: 0.33–0.48)

B: External evaluation of CDBRS models					
Model	**Mean AUC‐ROC**	**Generalized c‐index**	**AUC‐ROC**	**Specificity**	**Sensitivity**
CDBRS‐3* (NACC)	0.72 (95% CI: 0.69–0.74)	0.71 (95%CI: 0.69–0.72)	NA	NA	NA
CDBRS‐3* (ROSMAP)	0.70 (95% CI: 0.68–0.72)	0.70 (95% CI: 0.68–0.73)	NA	NA	NA
CDBRS‐2* (NACC)	NA	NA	0.71 (95% CI, 0.70–0.72)	0.70 (95% CI: 0.66–0.72)	0.73 (95% CI: 0.71–0.75)
CDBRS‐2* (ROSMAP)	NA	NA	0.70 (95% CI, 0.68–0.74)	0.69 (95% CI: 0.66–0.73)	0.74 (95% CI: 0.72–0.75)

*Note*: Sky blue, classification of low, medium, high risk; Grey, binary classification.

* Model using features available in both NACC and ROSMAP datasets.

Abbreviations: AUC, area under the receiver operating characteristics curve; CDBRS, Cerebrovascular Disease Burden Risk Score; CI, confidence interval; NACC, National Alzheimer's Coordinating Center; POM, Proportional‐odds Model; ROSMAP, Religious Orders Study and Memory and Aging Project.

### 3.5 External evaluation outcomes

We[Fig alz70543-fig-0003] employed the ROSMAP data for the external evaluation of the CDBRS models; however, the features recorded by ROSMAP and NACC do not align perfectly. In the ROSMAP cohort, data for the Boston Naming Test and heart rate were unavailable. Therefore, in the CDBRS‐3 model we substituted these features with years of education and MMSE score, the next highest‐ranked features available in ROSMAP. Smoking was not considered, as it was recorded differently between NACC and ROSMAP dataset. For CDBRS‐2, the external evaluation employed seven features, replacing heart rate with MMSE score. The model performance using ROSMAP data was comparable to that using NACC data (Table 3B). Overall, the external evaluation demonstrated that the CDBRS can achieve a good performance across independent datasets.

### 3.6 Epidemiology analysis

After adjusting for potential confounders, older age was significantly associated with increased odds of both microinfarcts (OR 1.06 [1.05–1.07]) and old infarcts (OR 1.04 [1.03–1.05]). Notably, compared to the normal and healthy weight, being underweight was associated with higher odds of hemorrhages/microbleeds (OR 2.80 [1.14–6.89]). Obesity was associated with higher odds of microinfarcts (OR 1.25 [1.02–1.54]) and old infarcts observed grossly (OR 1.53 [1.20–1.95]). Higher systolic blood pressure was also associated with greater odds of microinfarcts. Moreover, moderate and severe arteriolosclerosis was consistently linked to increased odds of hemorrhages/microbleeds, microinfarcts, and old infarcts. In addition, a lower CDR Sum of Boxes (CDR‐SB) score was significantly associated with higher odds of microinfarcts. These findings were detailed in the Table 4.

**TABLE 4 alz70543-tbl-0004:** Associations between participant characteristics and presence of microinfarcts hemorrhages/microbleeds, and grossly observed old infarcts.

Measures	Hemorrhages/microbleeds (ref: No)	Microinfarcts (ref: No)	Old infarcts observed grossly (ref: No)
**Demographic, lifestyle, genetic**			
Age (in years)	1.00 [0.99–1.01], *p* = 0.9791	**1.06 [1.05–1.07],** ***p* = 0.0001**	**1.04 [1.03–1.05],** ***p* = 0.0001**
Education (in years)	0.98 [0.93–1.03], *p* = 0.3448	1.00 [0.97–1.03], *p* = 0.9600	1.00 [0.97–1.04], *p* = 0.8530
Packs smoked			
1 cigarette to less than 0.5 pack	1.04 [0.69–1.58], *p* = 0.8557	0.80 [0.62–1.03], *p* = 0.0891	1.14 [0.85–1.51], *p* = 0.3824
0.5 pack to less than 1 pack	0.84 [0.55–1.27], *p *= 0.4020	1.00 [0.79–1.25], *p* = 0.9674	1.22 [0.93–1.59], *p *= 0.1439
1 pack to 1.5 packs	0.90 [0.52–1.54], *p *= 0.7002	**1.37 [1.02–1.84],** ***p* = 0.0349**	1.10 [0.76–1.59], *p *= 0.6054
0.5 packs to 2 packs	0.57 [0.23–1.42], *p *= 0.2246	0.92 [0.59–1.43], *p *= 0.7062	1.00 [0.58–1.70], *p* = 0.9899
More than two packs	1.29 [0.67–2.47], *p* = 0.4472	0.76 [0.48‐1.21], *P =* 0.2436	1.46 [0.91‐2.34], *P =* 0.1140
APOE ε4 carrier status (Yes/No)	0.90 [0.68‐1.19], *p *= 0.4620	0.85 [0.72–1.00], *p* = 0.050	0.92 [0.76–1.12], *p* = 0.3978
**Vascular and cardiovascular measures**			
BMI			
Under weight	**2.80 [1.14–6.89],** ***p* = 0.0246**	0.57 [0.26–1.26], *p* = 0.1647	1.28 [0.59–2.82], *p* = 0.5447
Overweight	1.09 [0.78–1.53], *p* = 0.6028	1.09 [0.89–1.32], *p* = 0.4055	1.21 [0.95–1.52], *p* = 0.1159
Obese	1.10 [0.77–1.57], *p* = 0.6152	**1.25 [1.02–1.54],** ***p* = 0.0303**	**1.53 [1.20–1.95],** ***p* = 0.0006**
Heart rate (pulse)	1.01 [0.99–1.02], *p* = 0.2385	1.00 [0.99–1.01], *p* = 0.7515	1.00 [0.99–1.01], *p* = 0.7081
Systolic blood pressure			
Elevated	0.83 [0.51–1.35], *p* = 0.4531	**1.67 [1.26–2.22],** ***p* = 0.0004**	1.08 [0.77–1.51], *p* = 0.6494
High stage 1	0.71 [0.46–1.10], *p* = 0.1280	**1.31 [1.01–1.70],** ***p* = 0.0397**	0.93 [0.69–1.25], *p* = 0.6271
High stage 2	0.98 [0.69–1.39], *p* = 0.9108	**1.42 [1.14–1.78],** ***p* = 0.0020**	1.11 [0.87–1.43], *p* = 0.3993
Diastolic blood pressure categories			
Elevated	0.81 [0.53–1.24], *p* = 0.3334	1.15 [0.90–1.45], *p* = 0.2591	0.97 [0.72–1.29], *p* = 0.8143
High	0.85 [0.58–1.25], *p* = 0.4064	1.12 [0.90–1.39], *p* = 0.3245	1.14 [0.88–1.47], *p* = 0.3102
Arteriolosclerosis			
Mild	1.38 [0.81–2.37], *p* = 0.2402	**2.02 [1.46–2.80],** *p* = **0.0001**	1.49 [1.01–2.20], *p* = 0.0429
Moderate	**1.80 [1.06–3.07],** ***p* = 0.0308**	**3.01 [2.18–4.15],** ***p* = 0.0001**	**2.55 [1.75–3.70],** ***p* = 0.0001**
Severe	**3.43 [1.97–5.98],** ***p* = 0.0001**	**4.16 [2.93–5.91],** ***p* = 0.0001**	**4.13 [2.77–6.16],** ***p* = 0.0001**
**Cognitive and functional measures**			
WAIS	1.00 [0.99–1.01], *p* = 0.9336	1.00 [0.99–1.00], *p* = 0.2622	1.00 [0.99–1.01], *p* = 0.7105
Animal	1.00 [0.97–1.02], *p* = 0.7280	1.01 [0.99–1.02], *p* = 0.2977	1.00 [0.99–1.02], *P =* 0.9323
GDS	1.03 [0.97–1.10], *p* = 0.3165	0.97 [0.94–1.01], *p* = 0.1594	1.02 [0.98–1.06], *p* = 0.4191
Boston	0.99 [0.97–1.01], *p* = 0.3532	1.01 [1.00–1.03], *p* = 0.0662	1.01 [0.99–1.02], *p* = 0.4452
MMSE	0.99 [0.97–1.02], *p* = 0.4824	1.01 [0.99–1.02], *p* = 0.4214	**1.02 [1.00–1.04],** *p* = **0.0471**
CDR‐SB	1.00 [0.96–1.04], *p* = 0.8075	**0.97 [0.95–0.99],** *p* = **0.0152**	0.99 [0.97–1.02], *p* = 0.6621

*Note*: Adjusted model, covariates included age (in years), sex, and APOE ε4 carrier status (yes/no). Statistically significant results (*p* < 0.05) are in bold.

Abbreviations: APOE, apolipoprotein E; BMI, body mass index; CDR‐SB, Clinical Dementia Rating–Sum of Boxes; CI, confidence interval; GDS, Geriatric Depression Scale; MMSE, Mini‐Mental State Examination; WAIS, Wechsler Adult Intelligence Scale.

## 4 DISCUSSION

In this study, we developed and validated the CDBRS, an interpretable machine‐learning model that stratifies cerebrovascular disease burden in older adults. Trained and validated on the NACC cohort and externally evaluated in the independent ROSMAP cohort. Several machine learning models have been developed and validated to predict the risk of ischemic and hemorrhagic stroke.^28^, ^29^, ^30^, ^31^ To our knowledge, this is the first study to predict the risk of cerebrovascular disease burden, specifically microbleeds, microinfarcts, and infarcts, using a machine learning model without relying on advanced imaging data as predictive features. Microbleeds, microinfarcts, and infarcts often precede clinical stroke and are considered early indicators of cerebrovascular disease progression. As such, their risk prediction holds significant clinical value for early intervention and prevention strategies. By integrating demographic, clinical, and neuropsychological data, the CDBRS‐3 effectively stratifies individuals into low‐, medium‐, and high‐risk groups, while the CDBRS‐2 provides binary risk classification. By predicting this occult cerebrovascular risk, the CDBRS aids clinical decision‐making by identifying high‐risk individuals who may benefit from closer monitoring or more intensive management of vascular risk factors to slow disease progression and prevent stroke or vascular dementia. Additionally, the model can guide therapeutic decisions—for example, a high CDBRS may warrant caution with treatments that carry hemorrhagic risk, whereas a low score may support the use of more intensive interventions. Ultimately, the CDBRS enhances clinical value by making an otherwise hidden risk visible, enabling more personalized prevention and treatment strategies to improve neurological outcomes.

Despite[Table alz70543-tbl-0003], [Table alz70543-tbl-0004] some demographic differences between the NACC and ROSMAP datasets, the CDBRS achieved an accurate prediction in ROSMAP comparable to that in NACC, underscoring its robustness and generalizability across study cohorts. The promising predictive performance is further illustrated by that the CDBRS models outperformed their respective baseline models. These results suggest that the AutoScore‐based feature selection and scoring approach used in CDBRS effectively captured complex data patterns that simpler models failed to detect. Furthermore, by leveraging the AutoScore framework, the final model achieved a strong predictive performance while maintaining practicality through a limited number of features.

Reviewing of the CDBRS scoring table (Table 2) shows that arteriolosclerosis‐severity, age, and BMI are the major contributors to the CDBRS score. The points assigned to arteriolosclerosis increase steadily with disease severity, aligning with the neuropathological evidence that greater arteriolosclerosis is linked to cerebrovascular risk factors.^32^ This observation is also in line with our epidemiologic analyses—moderate and severe arteriolosclerosis were associated with higher odds of cerebrovascular disease burden risk, whereas none or mild arteriolosclerosis corresponded to lower odds. Age also contributes substantially to the CDBRS score, which is in line with its strong association with microinfarctions and old infarcts shown in our epidemiological analysis. Furthermore, our epidemiological analysis also shows that BMI exhibits the anticipated U‐shaped association with cerebrovascular disease risk, with both underweight and obese individuals demonstrating higher odds, consistent with the fine‐tuning score table. On the other hand, interpretation of epidemiological analysis also reinforces the biological plausibility of the important predictive feature identified using AutoScore framework. Age and systolic blood pressure capture the cumulative hemodynamic stress that accelerates arteriolosclerosis,^33^ while current smoking signifies an exogenous vascular toxin^34^; together they map onto a cascade that yields microinfarcts and culminates in gross infarcts. Neuropsychological indices (e.g., CDR‐SB) likely reflect early functional footprints of this microvascular injury, explaining their selection despite indirect mechanistic ties. Arteriolosclerosis across all three risk levels supports its role as a convergent pathway rather than an isolated marker and justifies its weighted contribution in the score. Overall, the CDBRS remains an evidence‐based, clinically relevant instrument for grading disease severity.

A key strength of the CDBRS model lies in its direct solution to the “black box” challenge often associated with machine learning models. The lack of clinical interpretability in complex algorithms can significantly hinder their adoption.^35^ By using AutoScore, we transformed the underlying model into a transparent, point‐based score table. This makes each feature's contribution to the CDBRS immediately clear, which can be easily interpreted by clinicians. Epidemiological analysis was used to further validate the features selected by CDBRS. This confirmed a significant association between age, arteriosclerosis, and our outcomes. Interestingly, the machine learning techniques identified features that epidemiological analysis could not, indicating their potential as a more sensitive method. Moving forward, we advocate for close collaboration between machine learning experts and clinical epidemiologists to refine these models, ensuring that predictive accuracy, interpretability, and methodological rigor continue to advance in tandem. Flexibility in feature selection is another potential key strength of the CDBRS model. Some predictive features were not consistently collected/recorded by both NACC and ROSMAP, for example, Boston Naming Test and heart rate. However, interestingly, replacing these features with MMSE score and years of education, the next available most important features, did not compromise the performance of CDBRS, demonstrating the model's resilience to pragmatic data constraints. The flexibility afforded by the AutoScore and AutoScore‐Ordinal frameworks thus enhances the CDBRS's suitability for real‐world clinical deployment.

Several limitations of this study and the CDBRS model warrant consideration. The development and validation cohorts (NACC and ROSMAP) are both U.S.‐based research data, which may limit global generalizability. Participants in these studies tend to be highly educated and lack ethnic diversity. As a result, the CDBRS may not fully capture risk factor profiles or outcome manifestations that differ by ethnicity or socio‐demographic context. From a clinical standpoint, the CDBRS (like any risk model) provides probability of disease burden rather than a certainty; thus, it should complement, not replace, clinical judgment.^36^ We acknowledge that prospective clinical impact was not assessed in this study; it remains to be shown that interventions guided by CDBRS scoring will translate into improved patient outcomes. Moreover, due to data availability constraints, the development of the CDBRS model did not include biomarkers and imaging data, despite their potential to enhance the model's performance. This limitation should be addressed in future studies.

Future efforts for the CDBRS model will focus on enhancing its predictive power and clinical integration. One of the strategies is to include additional data sources such as magnetic resonance imaging (MRI)‐based cerebrovascular disease markers and emerging blood biomarkers to provide direct evidence of cerebrovascular damage and improve accuracy. Another important step involves validating and recalibrating the CDBRS across diverse populations, including non‐White individuals and varied geographic/clinical settings, to ensure universal applicability. This may involve adjusting the scoring system through the AutoScore framework for population‐specific risk factors. We also plan to test the CDBRS prospectively in clinical studies to evaluate its ability to predict real‐world outcomes like incident stroke or vascular dementia/cognitive decline, and to determine if using the CDBRS to guide interventions improves patient outcomes. For clinical integration, developing user‐friendly tools like smartphone apps or electronic health record plugins is essential,^37^ alongside providing targeted education and training for healthcare providers on interpreting and applying the score. Overall, these directions aim to strengthen the CDBRS's predictive capability, broaden its applicability, and demonstrate its real‐world utility in cerebrovascular health management.

In conclusion, this study presents the CDBRS, a new and interpretable model for stratifying the risk of microbleeds, microinfarcts, and infarcts in older adults. While further validation in diverse populations and prospective trials is necessary, the CDBRS marks a significant advancement in identifying at‐risk individuals for timely vascular risk management. It holds promise for integration into clinical practice, supporting personalized decision‐making and ultimately improving patient outcomes.

## CONFLICT OF INTEREST STATEMENT

The authors have no conflicts to report.

## CONSENT STATEMENT

Each Alzheimer's Disease Center contributed data to the National Alzheimer's Coordinating Center (NACC) employed its own protocol to obtain informed consent from participants or, when participants were unable to provide consent, from their next of kin, caregivers, or legal guardians. Written informed consent was obtained from all participants by Rush University. All participants have consented to allow secondary analysis of the de‐identified data.

## Supporting information



Supporting Information

Supporting Information
