# Supplementary material for: Development and validation of a predictive model to forecast the cerebrovascular risk burden in older people using the NACC and ROSMAP dataset
Source: Alzheimers Dement. 2025 Aug 12;21(8):e70543. doi: 10.1002/alz.70543 (PMC12340427; doi:10.1002/alz.70543)
Supplement: Supplementary file 1 — Supporting Information [file ALZ-21-e70543-s001.docx]

**Supplemental Material - Table of Content**

**(A)** **Supplementary methods**

Module 1: Feature Ranking

Module 2: Variable Transformation

Module 3: Score Derivation

Module 4: Model Selection and Optimization

Module 5: Fine-Tuning Cutoff Points

Module 6: Performance Evaluation

Data handling

**(B) eFigures**

eFigure 1: Architecture of CDBRS.

eFigure 2: Calibration plot of the CDBRS-3 model

eFigure 3: Feature ranking for the CDBRS-2 model.

eFigure 4: Parsimony analysis of the CDBRS-2 model.

**(C) eTables**

eTable 1: Feature explanation

eTable 2: Performance of CDBRS-3 model with other number of features

eTable 3: Fine-tuning score table for CDBRS-2.

**(A) Supplementary methods**

The AutoScore framework consists of six modules. For modules 1 to 5, data from 70% of the NACC participants (1433, training set) were used, while for module 6, data from the remaining 30% NACC participants (614, test set) were utilized. The training/test data split was randomly performed ten times. For each training set, a 10-fold cross-validation was performed for Modules 1-5. Steps of data handling are also provided here to ensure results reproducibility.

**Module 1: Feature Ranking**

To identify the most significant features for score generation, the ensemble machine learning algorithm Random Forest (RF) was utilized. RF works by integrating multiple decision trees, with each tree developed using a classification or regression methodology. This ensemble approach, which bases its final output on the combined results of all trees, grants RF robust resistance to overfitting. In classification tasks, the Gini index was applied to find optimal splits, effectively minimizing the likelihood of misclassification.

**Module 2: Variable Transformation**

After feature selection, the chosen variables underwent preprocessing and transformation. In this framework, variables with more than ten categories were treated as continuous. To better model nonlinear effects, these continuous variables were then converted into categorical ones, with a practical maximum of five categories. For this study, continuous variables were divided into four intervals at the 0th, 5th, 20th, 80th, 95th, and 100th percentiles of their total values. For simplicity, adjacent intervals that received the same score (in Module 3) were automatically merged.

**Module 3: Score Derivation**

Variable weights were calculated using the cumulative link model with a logit link function, a common method for ordinal outcomes. Let ***Y*** represent the ordinal outcome with ***J*** categories (1 to ***J***), and ***X*** denote the predictor variables. The formula for the logit of cumulative probabilities is:

$$P\left( \boldsymbol{Y}\leq j | \boldsymbol{X} \right)=\log\left( \frac{p_{j}}{1-p_{j}} \right)=\alpha_{j}-\boldsymbol{X}^{T}\boldsymbol{\beta}$$

where$\alpha_{j}$is the category-specific intercept and $\boldsymbol{\beta}$ represents the regression coefficients. To simplify interpretation, the model is adjusted to ensure all $\boldsymbol{\beta}$ values are positive. Coefficients are normalized relative to the smallest $\boldsymbol{\beta}$, and scores for each category are calculated as $\beta_{j}\left( score \right)=round\left( \frac{\beta_{j}}{\beta_{low}} \right)$. These scores form a table, with the total score determined by summing category points.

**Module 4: Model Selection and Optimization**

The model was optimized for both simplicity and predictive accuracy. To balance these objectives, a parsimony plot was utilized, which helped identify the optimal number of features where performance improvements began to plateau.

**Module 5: Fine-Tuning Cutoff Points**

The continuous variable cutoffs initially generated in Module 2 were refined for clinical relevance. This involved merging, rounding, or adjusting intervals to align with established medical norms and guidelines. Following these adjustments, Modules 2 and 3 were re-executed to ensure the final model maintained both robustness and clinical meaningfulness.

**Module 6: Performance Evaluation**

To assess its predictive accuracy, the model was evaluated using mean ROC curve analysis and Harrell’s generalized c-index (ROC curve analysis for binary). These robust metrics provide a comprehensive evaluation of the model's performance on unseen test data, specifically by gauging how effectively its predictions rank against observed outcomes, accounting for the presence of tied ranks.

**Data Handling**

This section outlines the steps taken to prepare and process the data for model development and evaluation.

**Step 1: Data Acquisition**

The NACC dataset (https://naccdata.org/) and the ROSMAP dataset (https://www.rushu.rush.edu/) were obtained through a formal application procedure.

**Step 2: Participant and Variable Selection**

Upon review, variables with more than 10% of missing data and less important to the target feature were excluded from the analysis. Only participants with complete records for the remaining variables were included for model development. The data was then randomly split into a training set (70%) and a test set (30%).

**Step 3: Model Development with AutoScore-Ordinal**

The six modules of the auto-score-ordinal algorithm were run using RStudio (version 12.0+369) to generate the CDBRS table. Ensure the AutoScore-Ordinal package (available at nliulab/AutoScore-Ordinal (github.com)) is installed in your R programming environment before execution.

**Step 4: Prediction and External Evaluation**

Finally, the generated CDBRS score table was applied to the test set. This step also served for external evaluation of the model's performance. The complete data handling process is visually summarized in **eFigure 1**.

**(B) eFigures**


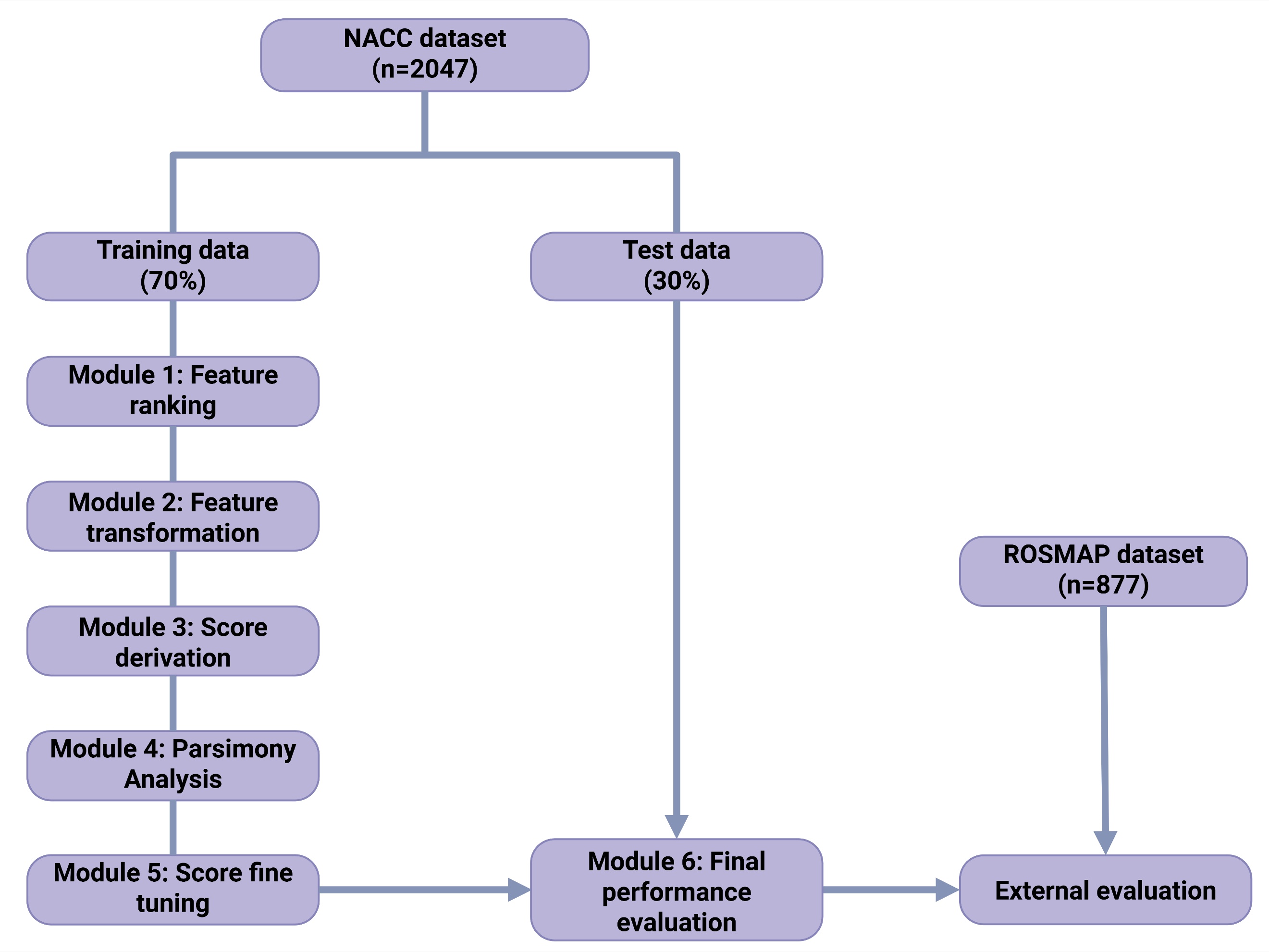


**eFigure 1: Architecture of CDBRS model**. The model is developed with NACC data, and external evaluation was performed using the ROSMAP data.


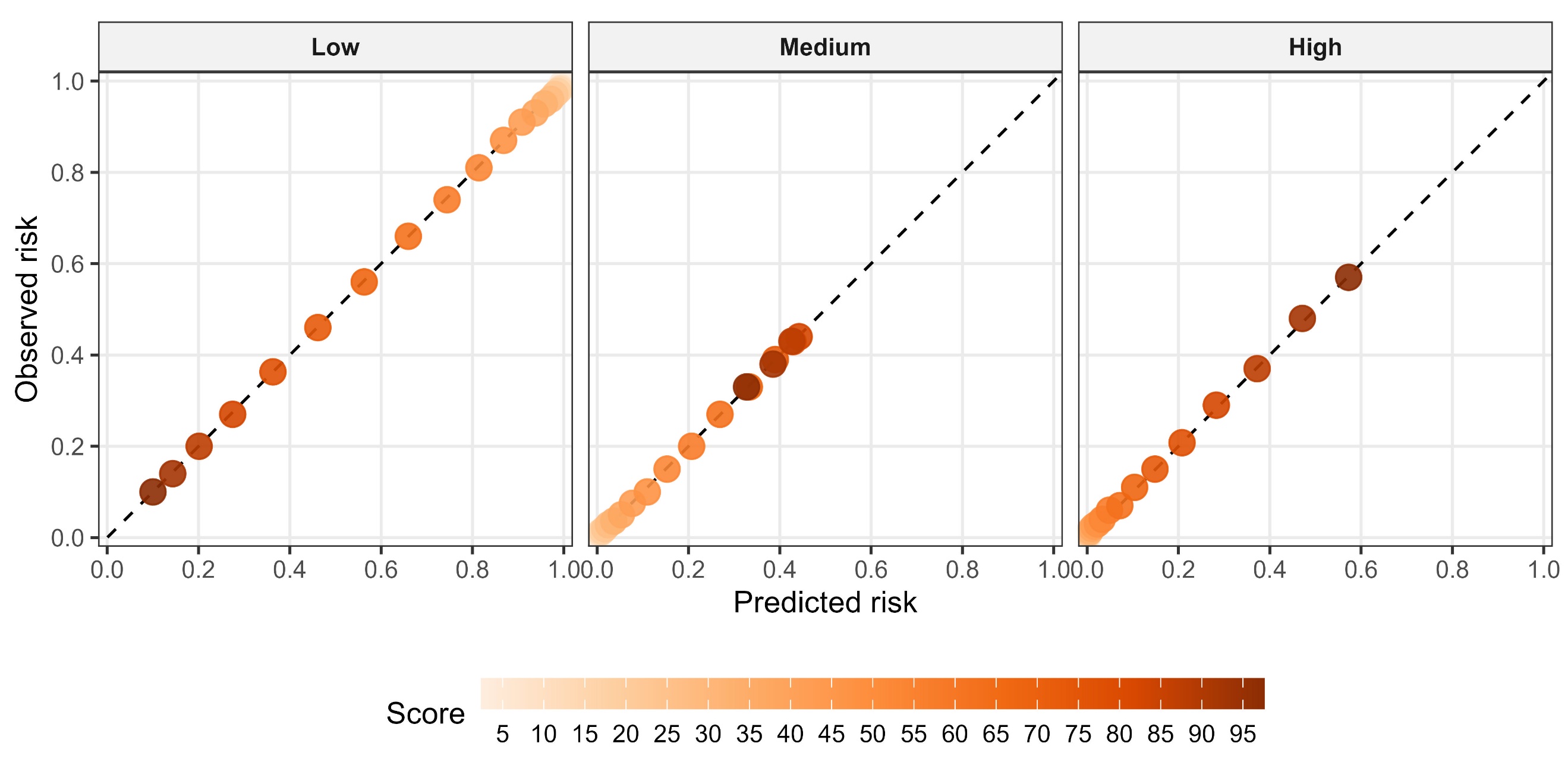


**eFigure 2: Calibration plot of the CDBRS-3 model.** Points represent 20 five-point score bands; color darkens with higher score. Panels separate low, medium, and high outcome categories. The dashed 45°-line marks perfect calibration—points above suggest over-estimation, below indicate under-estimation.


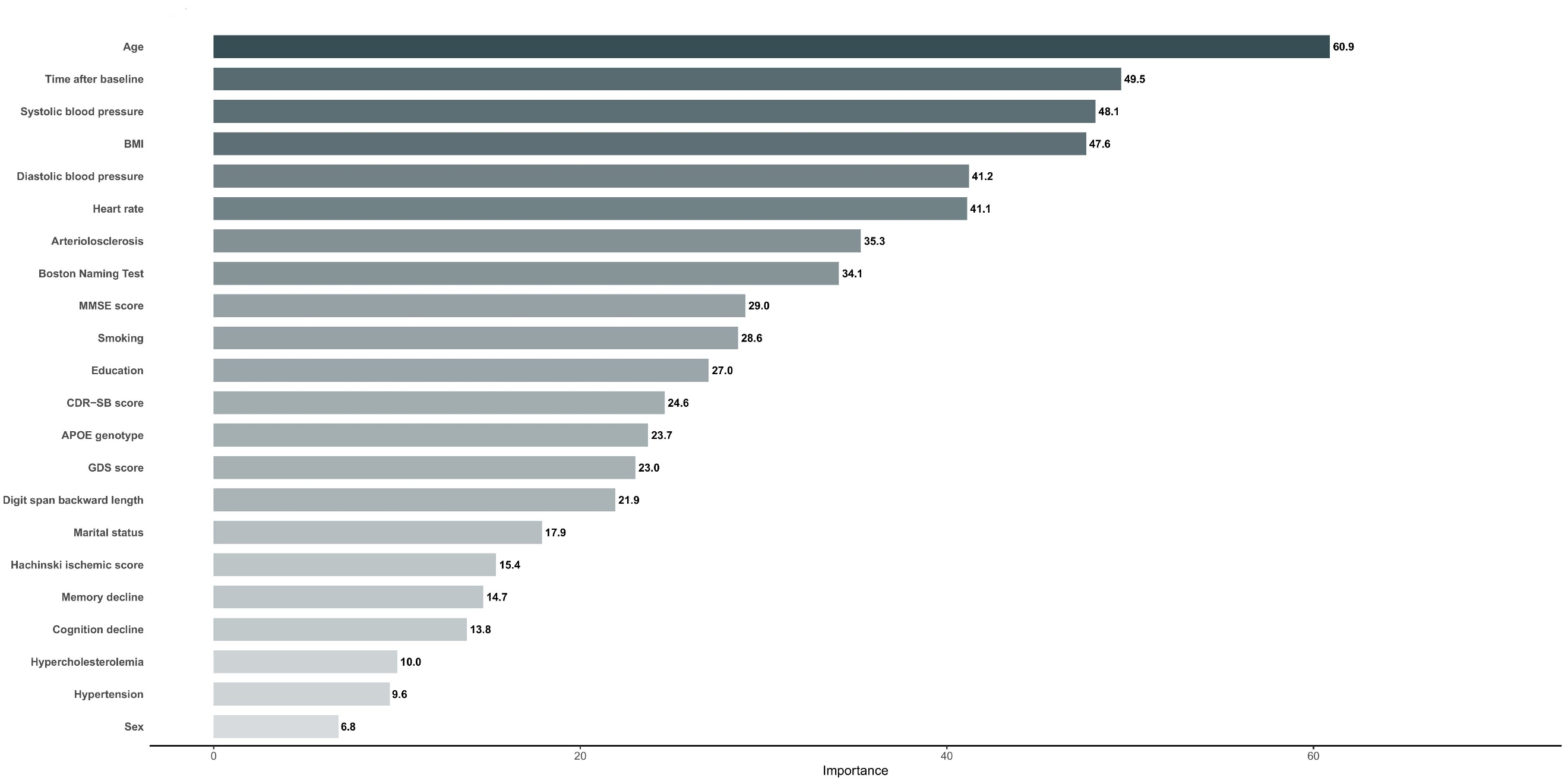


**eFigure 3: Feature ranking for the CDBRS-2 model.** The x-axis represents the importance of features as determined by random forest feature selection, while the y-axis lists the names of all features included in the study. Wider bars indicate higher importance.

**
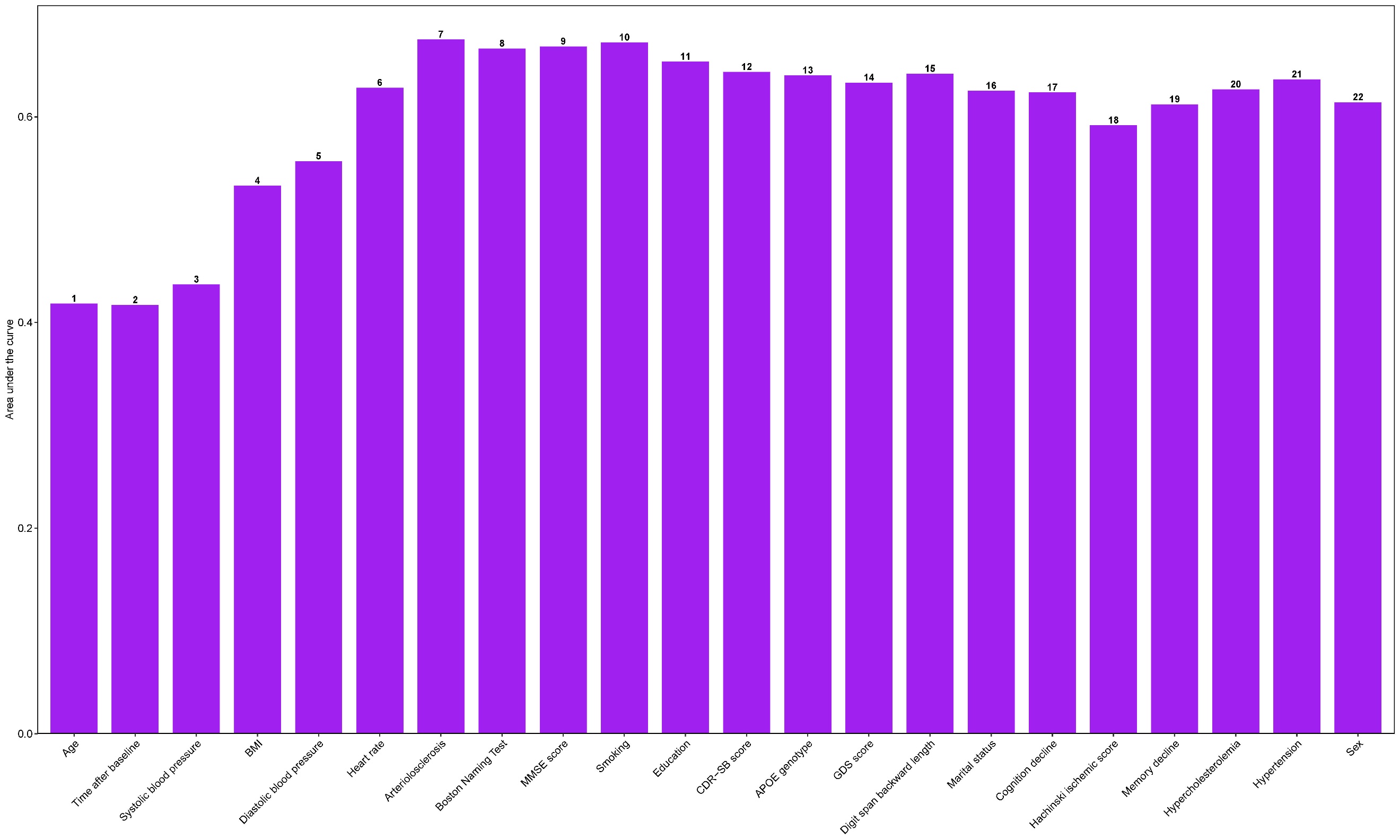
**

**eFigure 4: Parsimony analysis of the CDBRS-2 model.** This plot shows the average mean AUC-ROC values as the number of variables included in the CDBRS-2 model increases. The number annotated on each bar indicates the cumulative number of predictors used. Taller bars correspond to better model performance.

**eTable 1: Feature explanation**

| **Feature** | **Explanation** |
| --- | --- |
| ABRUPT | Abrupt onset |
| AGE | Age |
| ALCOHOL | Alcohol abuse |
| APOE | APOE genotype |
| BEAGIT | Subject currently manifests meaningful change in behavior -Agitation |
| BEAHALL | Subject currently manifests meaningful change in behavior – Psychosis, Auditory hallucinations |
| BEAPATHY | Subject currently manifests meaningful change in behavior -Apathy, withdrawal |
| BEDEL | Subject currently manifests meaningful change in behavior - Psychosis Abnormal, false, or delusional beliefs |
| BEDEP | Subject currently manifests meaningful change in behavior - Depressed mood |
| BEDISIN | Subject currently manifests meaningful change in behavior - Disinhibition |
| BEIRRIT | Subject currently manifests meaningful change in behavior - Irritability |
| BEMODE | Mode of onset of behavioral symptoms |
| BEPERCH | Subject currently manifests meaningful change in behavior - Personality change |
| BEVHALL | Subject currently manifests meaningful change in behavior - Psychosis Visual hallucinations |
| BMI | Body mass index |
| BOSTON | Boston Naming Test (30) - Total score |
| BPSYS | Subject blood pressure (sitting), systolic |
| CBTIA | Transient ischemic attack (TIA) |
| CDRSUM | CDR sum of boxes |
| CVAFIB | Atrial fibrillation |
| CVCHF | Congestive heart failure |
| CVHATT | Heart attack/cardiac arrest |
| DIABETES | Diabetes |
| DIGIBLEN | Digit span backward length |
| EDUC | Education |
| EMOT | Emotional incontinence |
| EVENTS | In the past four weeks, did the subject have any difficulty or need help with: Keeping track of current events |
| EYEMOVE | Are there eye movement abnormalities present indicative of central nervous system disorder? |
| GDS | GDS score |
| HACHIN | Hachinski ischemic score |
| HANDED | Is the subject left- or right-handed? |
| HRATE | Heart rate |
| HYPERCHO | Hypercholesterolemia |
| HYPERTEN | Hypertension |
| INDEPEND | Level of independence |
| MARISTAT | Marital status |
| NACCAPOE | APOE genotype |
| Arteriolosclerosis | Arteriolosclerosis severity |
| NACCCOGF | Indicate the predominant symptom that was first recognized as a decline in the subject’s cognition |
| NACCDAYS | Time after baseline |
| NACCLIVS | Living situation |
| NACCMMSE | Total MMSE score |
| NACCTBI | History of traumatic brain injury |
| PACKSPER | Average number of packs smoked per day |
| REMDATES | In the past four weeks, did the subject have any difficulty or need help with: Remembering appointments, family occasions, holidays, medications |
| RESIDENC | Type of residence |
| SEX | Sex of individual |
| THYROID | Thyroid disease |
| TRAUMBRF | Brain trauma with brief unconsciousness |
| VISCORR | Does the subject usually wear corrective lenses? |
| VISION | Without corrective lenses, is the subject’s vision functionally normal? |

**eTable 2: Performance of CDBRS-3 model with other number of features**

| **Number of features** | **Mean AUC-ROC** | **Generalized c-index** |
| --- | --- | --- |
| 9 | 0.69 (95%CI, 0.67-0.72) | 0.70 (95%CI, 0.69-0.74) |
| 10 | 0.70 (95%CI, 0.66-0.74) | 0.70 (95%CI, 0.68-0.73) |
| 11 | 0.71 (95%CI, 0.69-0.72) | 0.71 (95%CI, 0.69-0.73) |
| 12 | 0.71 (95%CI, 0.68-0.74) | 0.70 (95%CI, 0.69-0.75) |

**eTable 3: Fine-tuning score table for CDBRS-2.**

| Variable | Interval | Score |
| --- | --- | --- |
| Age* | <60 | 0 |
|  | [60,80) | 20 |
|  | ≥80 | 30 |
| Body mass index* | <18.5 | 2 |
|  | [18.5,25) | 0 |
|  | [25,30) | 4 |
|  | ≥30 | 5 |
| Time after baseline | <2.91 | 0 |
|  | [2.91,8.34) | 3 |
|  | ≥8.34 | 7 |
| Blood pressure  (systolic)* | <120 | 0 |
|  | [120,150) | 4 |
|  | >=150 | 10 |
| Arteriolosclerosis | None | 0 |
|  | Mild | 13 |
|  | Moderate | 27 |
|  | Severe | 32 |
| Heart rate* | <60 | 0 |
|  | [60,100) | 0 |
|  | ≥100 | 9 |
| Blood pressure  (diastolic)* | <60 | 4 |
|  | [60,90) | 2 |
|  | ≥90 | 0 |

* fine-tuning according to clinical norms
